# Pre- and post-diagnostic β-blocker use and lung cancer survival: A population-based cohort study

**DOI:** 10.1038/s41598-017-02913-8

**Published:** 2017-06-06

**Authors:** Janick Weberpals, Lina Jansen, Walter E. Haefeli, Michael Hoffmeister, Martin Wolkewitz, Myrthe P. P. van Herk-Sukel, Pauline A. J. Vissers, Hermann Brenner

**Affiliations:** 1 Division of Clinical Epidemiology and Aging Research, German Cancer Research Center (DKFZ), Heidelberg, Germany; 2 Department of Clinical Pharmacology and Pharmacoepidemiology, University Hospital of Heidelberg, Heidelberg, Germany; 3 Center for Medical Biometry and Medical Informatics, Institute for Medical Biometry and Statistics, Medical Center, University of Freiburg, Freiburg, Germany; 4 PHARMO Institute for Drug Outcomes Research, Utrecht, The Netherlands; 5 Netherlands Comprehensive Cancer Organisation, Utrecht, The Netherlands; 6 Division of Preventive Oncology, National Center for Tumor Diseases (NCT), German Cancer Research Center (DKFZ), Heidelberg, Germany; 7 German Cancer Consortium (DKTK), German Cancer Research Center (DKFZ), Heidelberg, Germany

## Abstract

Beta-blockers have been associated with decreased cancer mortality. However, evidence for lung cancer is sparse and reported beneficial effects might be based on biased analyses. In this so far largest study we investigated the association between β-blocker use and lung cancer survival. Therefore, patients with a lung cancer diagnosis between April 1998 and December 2011 were selected from a database linkage of the Netherlands Cancer Registry and the PHARMO Database Network. After matching eligible patients on the propensity score, adjusted hazard ratios (HRs) and corresponding 95% confidence intervals (CI) were calculated using Cox proportional hazards regression to investigate the association between pre-diagnostic and time-dependent β-blocker use and overall survival. Duration and dose-response analyses and stratified analyses by β-blocker type, histological subgroups and stage were conducted. Of 3,340 eligible lung cancer patients, 1437 (43%) took β-blockers four months prior to diagnosis. Pre-diagnostic β-blocker use was not associated with overall survival (HR 1.00 (0.92–1.08)) in the adjusted model. Time-dependent post-diagnostic analysis showed similar results with a HR of 1.03 (0.94–1.11). Trend analyses showed no association for cumulative dose (HR 0.99 (0.97–1.02)) and cumulative duration (HR 1.00 (0.96–1.05)). In conclusion, β-blocker use is not associated with reduced mortality among lung cancer patients.

## Introduction

With about 1.8 million new cases in 2012 lung cancer is the cancer type with the highest incidence worldwide^1^. Moreover, the prognosis for this disease still remains very poor and lung cancer represents the most common cause of death from cancer overall. Many previous experimental and epidemiological findings suggested that an upregulated activity of the sympathetic nervous system and cancer-related stress responses might lead to enhanced metastatic involvement and tumor growth which could be antagonized by β-adrenergic receptor blockade^2–4^.

Therefore, particularly β-blockers were proposed as a new add-on treatment for several cancer types. This hypothesis attracted much attention recently when propranolol, a nonselective β-blocker, was introduced as the new first-line treatment for infantile hemangiomas^5, 6^. However, so far only four observational studies^7–10^, with not more than a few hundred patients each, and two screening studies^11, 12^ investigated the association between β-blocker use and prognosis after lung cancer. Besides inconsistent results for non-small cell lung cancer (NSCLC), which were ranging from a protective to no association, no results have been published so far on small cell lung cancer (SCLC).

Additionally, the results of these studies are misleading as the analyses were found to be incorrect involving immortal time bias and insufficient confounder adjustment for potentially important prognostic factors^13, 14^.

Given that β-blockers are widely used for several indications and are considered as safe, effective, and well-established in routine care, benefits for lung cancer patients would be of utmost interest. Hence, these analyses aim to investigate the hypothesis whether concomitant β-blocker use is associated with a survival benefit among both NSCLC and SCLC patients. Including a population of 7,002 lung cancer patients, which is exceeding the study size of all previous studies taken together (N = 6,178), we provide results from the so far largest population-based study on the association between β-blocker use and lung cancer prognosis.

## Methods

### Data sources

The data used for this retrospective population-based cohort study comprises a comprehensive database linkage of the Netherlands Cancer Registry (NCR) and the PHARMO Database Network^15^. Data from the Eindhoven area of the NCR were used which covers a demographic region with 2.4 million inhabitants. The Eindhoven cohort of the NCR is a population-based cancer registry which collects information on patient and tumor characteristics, co-morbidities, and socio-economic status. Vital status is obtained by linkage to Dutch municipal records. The PHARMO Database contains longitudinal data gathered from community pharmacies including information on drug dispensing records, units and packages supplied, dose descriptions, and detailed information on active ingredients according to their Anatomical Therapeutic Chemical/Defined Daily Dose Classification (ATC/DDD) code^16^.

### Study population

Patients were eligible if they had a diagnosis of lung cancer between 1^st^ April 1998 and 31^st^ December 2011. Only primary lung cancers (with and without pathological confirmation) were included. Patients with missing information on important prognostic or risk factors were excluded. Due to the high model complexity of our statistical analyses and the sufficient amount of study participants, we refrained from using multiple imputation approaches to account for missing data. To mitigate healthy-user effects and confounding by indication we restricted the analysis cohorts to patients taking β-blockers or guideline medications prescribed alternatively to β-blockers during the four-month period prior to diagnosis (active comparators) as this was shown to lead to more unbiased results (Supplementary Table 1)^17^. Out of this population, a propensity score matched cohort was created to conduct all analyses in a quasi-experimental cohort setting, simulating equally distributed baseline factors.

### Classification and modelling of medication use

Patients were classified as β-blocker users if they received at least one β-blocker dispensing from the ATC code group C07 in a four-month interval. This interval was chosen, because an explorative analysis of the dataset has shown a more accurate representation of time-dependent drug utilization compared to a usually used three-month interval.

Beta-blocker subgroups according to their β_1_-receptor affinity (selective, nonselective) and single active ingredients were determined for subgroup analyses. As β-blockers were also shown to have variable tissue availability, subgroup analyses were additionally performed stratified by their pharmacokinetic properties (hydrophilicity/lipophilicity)^18^. To adjust for potential confounding effects by other medication classes, dispensed non-steroidal anti-inflammatory drugs (NSAIDs), statins, diabetes medications, antihypertensive treatments, and other medications with indications similar to those for β-blockers were also considered (Supplementary Table 1).

Use of β-blockers was once investigated as pre-diagnostic use and once as a time-varying covariate in post-diagnostic analyses. For pre-diagnostic analyses, β-blocker use was modelled as a time-fixed covariate, classifying patients as pre-diagnostic users if they received at least one β-blocker dispensing four months prior to diagnosis (yes/no). To overcome immortal time bias, the use of β-blockers after lung cancer diagnosis (post-diagnostic use) was modelled as a time-varying covariate according to the Mantel-Byar method, that is, patients were initially considered non-users and then users after a lag of four months after their first post-diagnostic β-blocker dispensing until death or end of follow-up^19–21^. To avoid time-varying confounding and selection bias a first-treatment-carried-forward approach was used to model treatment changes (intention-to-treat analysis)^22, 23^. The additional lag of four months was introduced to mitigate possible sick-stopper effects and to account for a biologically reasonable latency window and reverse causality^22, 24^. Given the information from the cancer registries that missing information on comorbidity will probably be more often seen among those without comorbidity, we additionally conducted sensitivity analyses coding missing information as having no comorbidity.

In post-diagnostic analyses, cumulative duration and cumulative dose were also investigated time-dependently starting with at least one dispensing four months prior to diagnosis. Cumulative duration of use was defined according to the following categories: 1–12 months, 13–24 months, 25–36 months and >36 months. The DDD of each dispensing was calculated by multiplying the dispensed number of tablets by the dose, divided by the DDD classification of the World Health Organization^16^ and were then categorized as 0 DDDs, >0–365 DDDs and 366+ DDDs. If doses were missing the dose from the previous treatment interval was used.

### Statistical analysis

The distribution of the basic characteristics of all eligible patients and the propensity score matched patients were compared between β-blocker users and active comparators based on β-blocker use four months prior diagnosis applying Pearson’s χ^2^ test.

The propensity score matched cohort was created by using logistic regression to calculate propensity scores, that is, the probability (propensity) of an individual receiving the treatment based on the patient’s observed time-invariant pre-treatment baseline variables as proposed by Rosenbaum and Rubin^25^. The following potential baseline prognostic and risk factors were included in the model: age, sex, year of diagnosis, socio-economic status (low, middle, high, institutionalized), comorbidities (cardiovascular, hypertension, cerebrovascular, lung, diabetes), treatment (surgery, chemotherapy, radiotherapy, radiotherapy aimed at metastasis), best supportive care, stage (UICC), histology (NSCLC, SCLC, other), previous cancer, baseline medication use and number of distinct ATC classes prescribed during four months prior to diagnosis^26^. Individuals were subsequently matched using a 1:1 nearest-neighbor matching algorithm with a caliper width of 0.2 standard deviations of the propensity score logit and without replacement as suggested by simulation studies^27–29^. Nearest neighbor matching was performed using the %*match_NearestNeighborMatch macro* provided by Rassen and Schneeweiss *et al*.^30^. To account for the matched nature of the sample (clustering in matched pairs), a robust (sandwich co-) variance (matrix) estimator that accounts for the clustering within matched sets was used^31, 32^.

Adjusted HRs, estimated by Cox proportional hazard regression, were used to assess the association between pre- and post-diagnostic β-blocker use and overall survival. Time-varying use of concomitant medication after diagnosis was included in the model to adjust for time-dependent effects. To account for the matched nature of the sample, a robust variance estimator that accounts for the clustering within matched sets was used^31, 32^. Follow up time was calculated using the reverse Kaplan-Meier method from date of diagnosis until death, migration from the NCR-PHARMO catchment area or end of study period (31^st^ December 2013), whichever occurred first.

Subgroup analyses were conducted by β-blocker subtype, histological subtypes and stage. As none of the previous studies considered active comparators as their reference group and propensity score matching in their analysis, we repeated all analyses with non-users as comparison group and conventional Cox regression without propensity score matching. Results from these analyses (presented in Supplementary Tables) have to be interpreted with caution, however, as they might be more prone to bias^17^.

The proportional hazards assumption was assessed by including a time-dependent component for each explanatory variable in the Cox model. For pre- and post-diagnostic use, the proportional hazards assumption was violated by treatment and stage covariates. As HRs did not meaningfully change when allowing these parameters for time-varying effects, we refrained from including these factors in the main analyses to keep the model complexity low.

All analyses were performed with SAS software, version 9.4 (SAS Institute Inc., Cary, NC, USA) according to an a-priori defined study protocol. Statistical significance was defined by a two-sided P < 0.05.

### Data availability

The dataset analyzed during the current study is a database linkage which is not publicly available and is licensed to be analyzed for the investigated research question only.

## Results

### Study population and medication use at baseline

**Figure 1:**
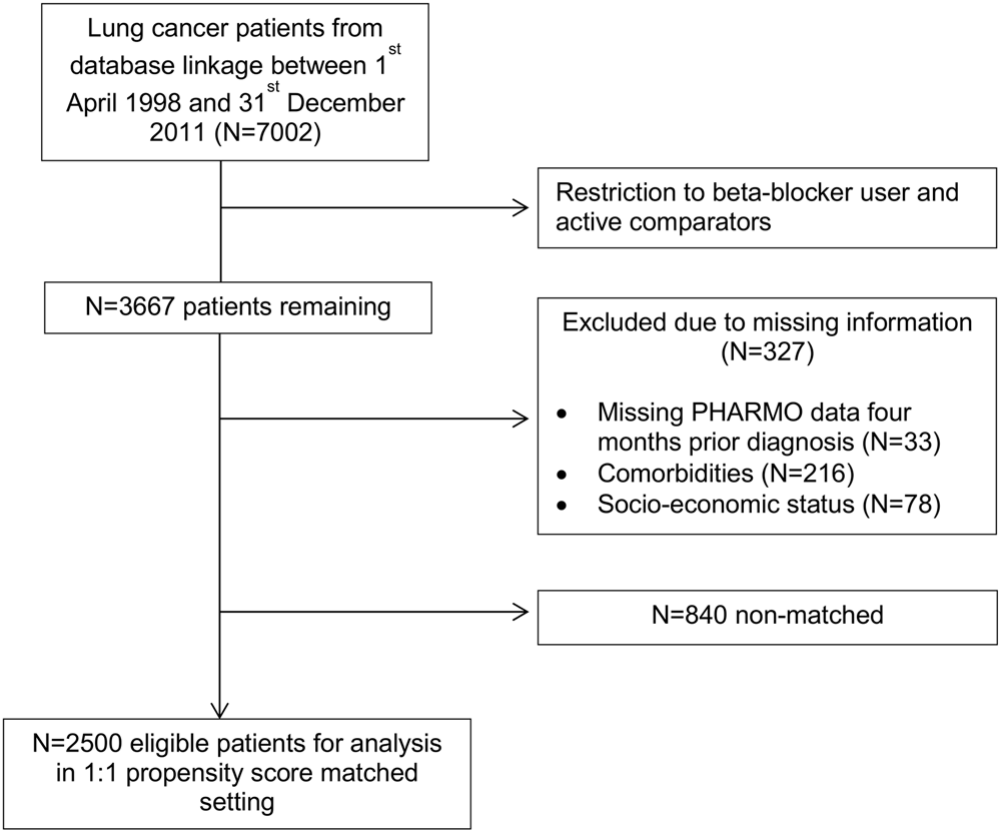
Selection of eligible patients for statistical analysis.

Out of 7002 lung cancer patients, 3340 patients (47.7%) were eligible to be included in the study either as β-blocker user or active comparator (Fig. 1).

The majority of patients were male (73%), diagnosed with NSCLC (71%) and had TNM stage III or IV (66%). Median follow-up time was 6.5 years (25th percentile: 4.1 years, 75th percentile: 9.5 years). During follow-up 2967 (89%) patients died, 2079 (70%) of them within the first 12 months.

Within the four months prior to diagnosis, 1437 (43%) of all patients took β-blockers. Selective β-blockers were used more frequently (38%) than nonselective β-blockers (5%) among all eligible patients. Metoprolol was the active ingredient used most (20%), followed by bisoprolol and atenolol (each 8%) and sotalol (3%). Restricting the analysis cohort to active comparators (before propensity score matching) already resulted in a more balanced cohort than simply comparing to non-users (Table 1). Βeta-blocker user and active comparators only differed significantly in the year of diagnosis, in the presence of comorbidities, use of other antihypertensives, use of statins and number of distinct medication classes dispensed.

**Table 1:** Baseline Characteristics of Lung Cancer Patients by Pre-diagnostic β-blocker Intake^a^.

Characteristics	All eligible patients (N = 3340)			PS matched analysis cohort^b^ (N = 2500)		
	β-blocker (N = 1437)	Comparator (N = 1903)	P	β-blocker (N = 1250)	Comparator (N = 1250)	P
**Age at diagnosis**						
30–59 years	165 (11.5%)	215 (11.3%)	0.7788	144 (11.5%)	151 (12.1%)	0.9678
60–69 years	455 (31.7%)	635 (33.4%)		405 (32.4%)	397 (31.8%)	
70–79 years	604 (42.0%)	779 (40.9%)		515 (41.2%)	514 (41.1%)	
80 + years	213 (14.8%)	274 (14.4%)		186 (14.9%)	188 (15.0%)	
Mean	70.5	70.2		70.4	70.4	
**Sex**						
Male	1059 (73.7%)	1379 (72.5%)	0.4277	917 (73.4%)	880 (70.4%)	0.0998
Female	378 (26.3%)	524 (27.5%)		333 (26.6%)	370 (29.6%)	
**Histology**						
NSCLC	1027 (71.5%)	1340 (70.4%)	0.2832	882 (70.6%)	898 (71.8%)	0.6060
SCLC	158 (11.0%)	243 (12.8%)		228 (18.2%)	209 (16.7%)	
Other	252 (17.5%)	320 (16.8%)		140 (11.2%)	143 (11.4%)	
**Year of diagnosis**						
1999–2001	111 (7.7%)	267 (14.0%)	<0.0001	109 (8.7%)	124 (9.9%)	0.6920
2002–2004	235 (16.4%)	369 (19.4%)		215 (17.2%)	207 (16.6%)	
2005–2007	416 (28.9%)	520 (27.3%)		360 (28.8%)	369 (29.5%)	
2008–2011	675 (47.0%)	747 (39.3%)		566 (45.3%)	550 (44.0%)	
**Socio-economic status**						
Low	519 (36.1%)	631 (33.2%)	0.1241	438 (35.0%)	452 (36.2%)	0.6038
Middle	572 (39.8%)	760 (39.9%)		497 (39.8%)	504 (40.3%)	
High	296 (20.6%)	424 (22.3%)		266 (21.3%)	256 (20.5%)	
Institutionalized	50 (3.5%)	88 (4.6%)		49 (3.9%)	38 (3.0%)	
**Stage at diagnosis (UICC)** ^**c**^						
I	239 (16.6%)	295 (15.5%)		197 (15.8%)	197 (15.8%)	0.9853
II	79 (5.5%)	103 (5.4%)		68 (5.4%)	70 (5.6%)	
III	411 (28.6%)	544 (28.6%)		371 (29.7%)	360 (28.8%)	
IV	549 (38.2%)	700 (36.8%)		469 (37.5%)	471 (37.7%)	
Surgery	251 (17.5%)	330 (17.3%)	0.9243	215 (17.2%)	219 (17.5%)	0.8327
Chemotherapy	501 (34.9%)	669 (35.2%)		440 (35.2%)	427 (34.2%)	0.5849
Radiotherapy	386 (26.9%)	541 (28.4%)	0.3166	340 (27.2%)	346 (27.7%)	0.7880
**Comorbidity at cancer diagnosis**						
Previous cancer	216 (15.0%)	283 (14.9%)	0.8978	189 (15.1%)	197 (15.8%)	0.6579
Cardiovascular disease	1003 (69.8%)	1025 (53.9%)	<0.0001	818 (65.4%)	808 (64.6%)	0.6749
Cerebrovascular disease	119 (8.3%)	212 (11.1%)	0.0062	114 (9.1%)	111 (8.9%)	0.8339
Diabetes	281 (19.6%)	321 (16.9%)	0.0455	240 (19.2%)	232 (18.6%)	0.6827
Hypertension	636 (44.3%)	598 (31.4%)	<0.0001	488 (39.0%)	495 (39.6%)	0.7744
Use of other antihypertensives	1009 (70.2%)	1219 (64.1%)	0.0002	853 (68.2%)	849 (67.9%)	0.8637
Use of NSAIDs	767 (53.4%)	996 (52.3%)	0.5524	661 (52.9%)	677 (54.2%)	0.5211
Use of statins	759 (52.8%)	790 (41.5%)	<0.0001	621 (49.7%)	601 (48.1%)	0.4236
Use of diabetes medication	247 (17.2%)	286 (15.0%)	0.0915	213 (17.0%)	202 (16.2%)	0.5543
**Number of distinct ATC classes**						
0	0 (0.0%)	0 (0.0%)	<0.0001	0 (0.0%)	0 (0.0%)	0.8949
1–3	206 (14.3%)	383 (20.1%)		200 (16.0%)	193 (15.4%)	
4–5	550 (38.3%)	740 (38.9%)		488 (39.0%)	485 (38.8%)	
6+	681 (47.4%)	780 (41.0%)		562 (45.0%)	572 (45.8%)	

Abbreviations: ATC = Anatomical Therapeutic Chemical Code, NSAIDs = Non-steroidal anti-inflammatory drugs, NSCLC = Non-small cell lung cancer, PS = Propensity score, SCLC = Small-cell lung cancer, UICC = Union Internationale Contre le Cancer.

^a^Use of medications is defined as having at least one dispensing during four months prior to diagnosis.

^b^Propensity scores (PS) were calculated using logistic regression. Individuals were matched using a 1:1 nearest neighbor matching algorithm with a caliper width of 0.2 standard deviations of PS logit and without replacement.

^c^Stage was not applicable or determinable for 400 patients (non-matched cohort) and 297 patients (propensity score matched cohort).

After matching users and active comparators on propensity scores, 2500 patients remained for statistical analysis (Fig. 1). The assessment of distributions of baseline variables showed that the propensity score matching led to a well-balanced cohort for all measured baseline characteristics (Table 1).

### Pre-diagnostic β-blocker use and survival

**Figure 2:**
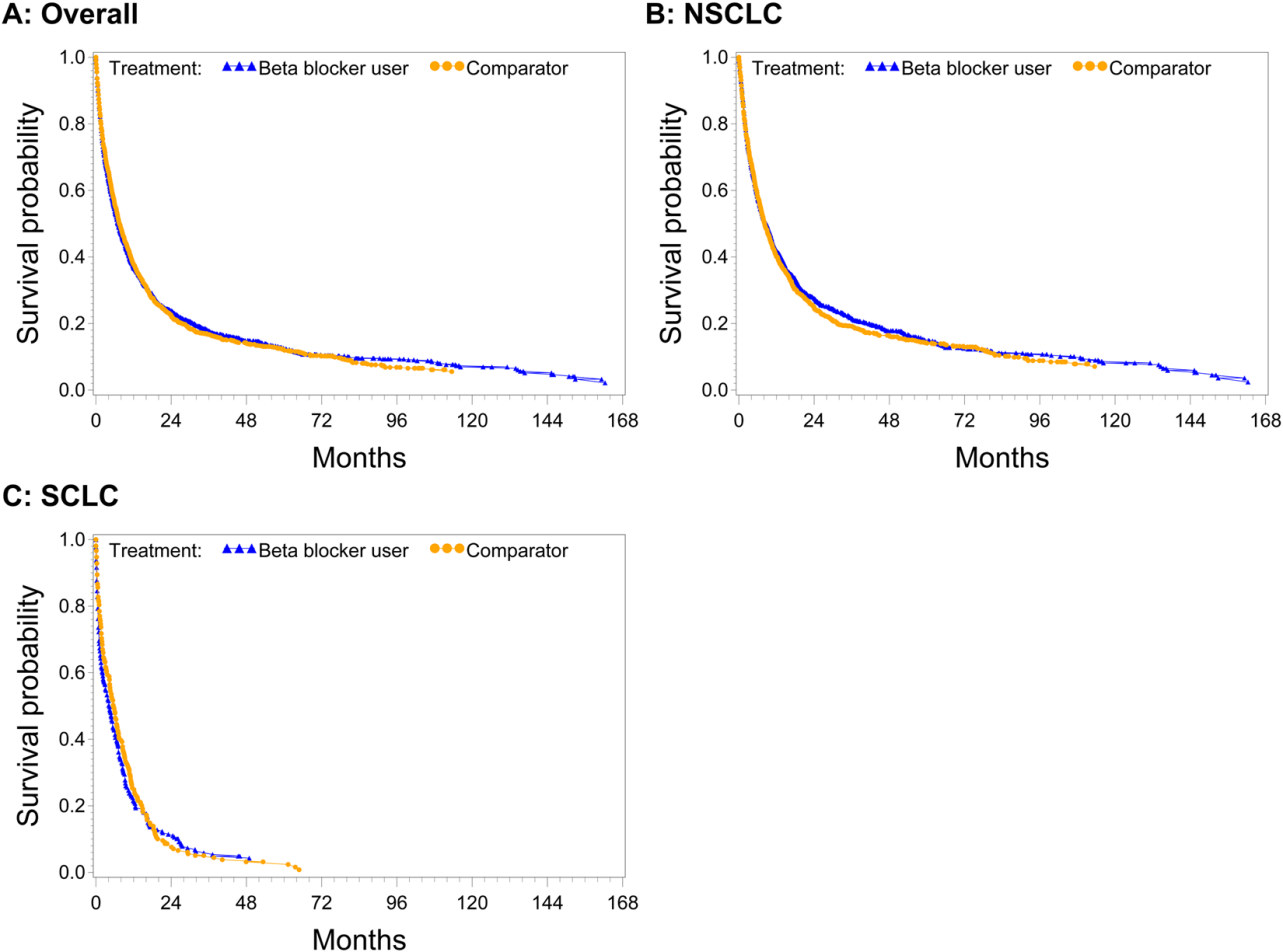
Adjusted survival curves for any β-blocker use in the four month interval prior diagnosis for (**A**) all histologies, (**B**) non-small cell lung cancer (NSCLC) and (**C**) small cell lung cancer (SCLC).

After adjustment for relevant covariates in multivariable analyses, no associations were observed for any β-blocker use (1.00 (0.92–1.08)) (Table 2 and Fig. 2).

**Table 2:** Association between Pre-diagnostic β-blocker use and Lung Cancer Survival (Overall, by Cancer Site and Cancer Stage).

β-blocker	Propensity score matched cohort (N = 2500)					
	Subgroup	N	Events	HR^a^	95% CI	P
No β-blocker						
	**Total**	1250	1114	1.00	Ref.	/
	**Stage I**	197	120	1.00	Ref.	/
	**Stage II**	70	57	1.00	Ref.	/
	**Stage III**	360	337	1.00	Ref.	/
	**Stage IV**	471	461	1.00	Ref.	/
	**NSCLC**	898	780	1.00	Ref.	/
	**SCLC**	209	204	1.00	Ref.	/
Any β-blocker						
	**Total**	1250	1107	1.00	0.92–1.08	0.9950
	**Stage I**	197	122	0.95	0.74–1.23	0.7157
	**Stage II**	68	55	0.89	0.60–1.31	0.5529
	**Stage III**	371	333	0.87	0.75–1.02	0.0822
	**Stage IV**	469	466	**1.20**	**1.06–1.35**	**0.0050**
	**NSCLC**	882	763	0.97	0.88–1.07	0.5161
	**SCLC**	228	216	1.04	0.86–1.25	0.6844
Selective β-blocker						
	**Total**	1112	982	0.97	0.89–1.05	0.4661
	**Stage I**	177	111	0.99	0.77–1.27	0.9123
	**Stage II**	60	48	0.86	0.57–1.29	0.4650
	**Stage III**	333	298	**0.85**	**0.73–0.99**	**0.0426**
	**Stage IV**	414	411	**1.18**	**1.04–1.34**	**0.0099**
	**NSCLC**	783	677	0.96	0.87–1.06	0.4416
	**SCLC**	207	195	0.96	0.79–1.15	0.6371
Nonselective β-blocker						
	**Total**	151	138	**1.22**	**1.01–1.46**	**0.0357**
	**Stage I**	21	12	0.90	0.47–1.74	0.7614
	**Stage II**	8	7	1.11	0.52–2.37	0.7807
	**Stage III**	45	42	1.21	0.87–1.67	0.2622
	**Stage IV**	58	58	1.10	0.85–1.42	0.4630
	**NSCLC**	108	95	1.08	0.87–1.34	0.4982
	**SCLC**	22	22	**1.67**	**1.14–2.46**	**0.0092**
Hydrophilic β-blocker						
	**Total**	298	264	0.97	0.85–1.11	0.7041
	**Stage I**	44	32	1.35	0.92–1.96	0.1208
	**Stage II**	22	21	1.19	0.74–1.92	0.4664
	**Stage III**	78	64	0.79	0.59–1.05	0.1026
	**Stage IV**	112	112	1.14	0.94–1.38	0.1812
	**NSCLC**	195	171	0.99	0.85–1.17	0.9421
	**SCLC**	60	56	1.02	0.75–1.39	0.8959
Lipophilic β-blocker						
	**Total**	935	828	1.02	0.93–1.11	0.6928
	**Stage I**	151	89	0.85	0.65–1.10	0.2097
	**Stage II**	45	33	0.75	0.49–1.15	0.1824
	**Stage III**	290	266	0.97	0.83–1.13	0.6793
	**Stage IV**	347	344	1.15	1.01–1.31	0.0312
	**NSCLC**	674	581	0.98	0.88–1.09	0.7143
	**SCLC**	164	156	0.98	0.81–1.20	0.8774

Abbreviations: ATC = Anatomical Therapeutic Chemical Code, CI = Confidence interval, HR = Hazard ratio, NSCLC = Non-small cell lung cancer, PY = Person-years, SCLC = Small cell lung cancer

^a^Hazard ratios from Cox proportional hazard regression on the propensity score matched groups for β-blocker use four months prior to diagnosis with additional adjustment for time-dependent use of Non-steroidal anti-inflammatory drugs, statins, antihypertensive (other than β-blocker) and diabetes medication after diagnosis. Stratification factors were omitted from the stratified models.

For β-blocker subgroups there were significant associations for nonselective β-blocker use (1.22 (1.01–1.46)). Analyses stratified by stage and site showed significant associations for stage III patients using selective β-blockers (0.85 (0.73–0.99)). However, HRs for stage IV for any β-blocker use, selective β-blocker use and SCLC in the nonselective β-blocker group were increased (1.20 (1.06–1.35), 1.18 (1.04–1.34) and 1.67 (1.14–2.46), respectively).

When repeating the analysis with non-users as reference group, HRs were partially higher but followed the same trend (0.99 (0.92–1.07) and 1.00 (0.93–1.09) for any β-blocker use) (Supplementary Table 2).

### Post-diagnostic β-blocker use and survival

The results for β-blocker use modelled as a time-varying covariate in time-dependent analyses are presented in Table 3.

**Table 3:** Association between Post-diagnostic β-blocker use and Lung Cancer Survival (Overall, by Histological Subtype and Cancer Stage).

β-blocker	Propensity score matched cohort (N = 2500)					
	Subgroup	PY	Events	HR^a^	95% CI	P
No β-blocker						
	**Total**	1714	997	1.00	Ref.	/
	**Stage I**	558	93	1.00	Ref.	/
	**Stage II**	146	53	1.00	Ref.	/
	**Stage III**	426	290	1.00	Ref.	/
	**Stage IV**	357	438	1.00	Ref.	/
	**NSCLC**	1316	694	1.00	Ref.	/
	**SCLC**	186	186	1.00	Ref.	/
Any β-blocker						
	**Total**	2452	1224	1.03	0.94–1.11	0.5509
	**Stage I**	935	149	1.01	0.78–1.32	0.9408
	**Stage II**	199	59	0.88	0.60–1.28	0.4948
	**Stage III**	695	380	0.94	0.80–1.10	0.4372
	**Stage IV**	349	489	**1.17**	**1.03–1.33**	**0.0132**
	**NSCLC**	1953	849	0.98	0.89–1.09	0.7633
	**SCLC**	256	234	1.11	0.92–1.34	0.2781
Selective β-blocker						
	**Total**	2237	1095	0.99	0.91–1.08	0.8902
	**Stage I**	849	137	1.06	0.82–1.37	0.6545
	**Stage II**	177	53	0.89	0.60–1.32	0.5561
	**Stage III**	640	346	0.93	0.79–1.09	0.3509
	**Stage IV**	309	432	**1.15**	**1.01–1.30**	**0.0304**
	**NSCLC**	1764	764	0.99	0.89–1.10	0.8310
	**SCLC**	242	210	1.00	0.83–1.21	0.9860
Nonselective BB						
	**Total**	302	170	**1.26**	**1.06–1.49**	**0.0071**
	**Stage I**	114	18	1.01	0.60–1.70	0.9719
	**Stage II**	27	9	1.19	0.60–2.35	0.6174
	**Stage III**	95	56	1.20	0.90–1.59	0.2183
	**Stage IV**	43	63	1.14	0.89–1.45	0.2977
	**NSCLC**	270	116	1.09	0.89–1.32	0.4068
	**SCLC**	18	29	**1.79**	**1.27–2.51**	**0.0009**
Hydrophilic β-blocker						
	**Total**	610	291	1.01	0.89–1.14	0.9193
	**Stage I**	174	36	1.33	0.94–1.88	0.1086
	**Stage II**	56	21	1.19	0.74–1.92	0.4664
	**Stage III**	182	79	0.87	0.67–1.14	0.3214
	**Stage IV**	79	115	1.15	0.95–1.38	0.1473
	**NSCLC**	433	189	1.01	0.87–1.19	0.8584
	**SCLC**	78	61	1.06	0.79–1.43	0.6994
Lipophilic β-blocker						
	**Total**	1939	949	1.03	0.95–1.12	0.4979
	**Stage I**	793	121	0.95	0.73–1.23	0.6987
	**Stage II**	146	39	0.78	0.52–1.16	0.2156
	**Stage III**	554	311	1.02	0.87–1.19	0.8032
	**Stage IV**	266	369	1.12	0.99–1.27	0.0808
	**NSCLC**	1588	672	0.99	0.90–1.10	0.8836
	**SCLC**	189	174	1.04	0.86–1.27	0.6659

Abbreviations: ATC = Anatomical Therapeutic Chemical Code, CI = Confidence interval, HR = Hazard ratio, NSCLC = Non-small cell lung cancer, PY = Person-years, SCLC = Small cell lung cancer.

^a^Hazard ratio from Cox proportional hazard regression on the propensity score matched groups for time-dependent β-blocker use after diagnosis with additional adjustment for time-dependent use of Non-steroidal anti-inflammatory drugs, statins, antihypertensive (other than β-blocker) and diabetes medication after diagnosis. Stratification factors were omitted from the stratified models.

Overall there was again no evidence for an association for any β-blockers (1.03 (0.94–1.11)). There was also no significant effect amongst all β-blocker subgroups besides an increase in mortality for nonselective β-blockers (1.26 (1.06–1.49)).

In stage and site specific analyses almost all results were clustered around the null. There was just a slight decrease in mortality for stage IV patients taking any (1.17 (1.03–1.33)) or selective (1.15 (1.01–1.30)) β-blocker and SCLC patients taking nonselective β-blocker (1.79 (1.27–2.51)).

When repeating the analysis without active comparison, estimates were again higher leading to a slight increase in hazard ratios for all eligible patients (1.09 (1.02–1.17)) while in a propensity score setting with non-users as reference there was still no significant association (1.03 (0.95–1.12)) (Supplementary Table 3).

### Cumulative duration and cumulative dose

Results from cumulative dose-response and duration analyses are shown in Table 4.

**Table 4:** Association between Post-diagnostic Cumulative Dose and Cumulative Duration of β-blocker use and Overall Lung Cancer Survival (Overall, by β-receptor Affinity and Pharmacokinetic Characteristics).

β-blocker	Propensity score matched cohort (N = 2500)					
	Dose/Duration	PY	Events	HR^a^	95% CI	P
Any β-blocker						
	**0 DDDs**	1651	991	1.00	Ref.	/
	**>0–365 DDDs**	1402	952	1.03	0.94–1.12	0.5273
	**366+ DDDs**	828	206	1.03	0.88–1.22	0.6899
	**Trend (180 DDDs)**			0.99	0.97–1.02	0.7006
	**0 months**	1714	997	1.00	Ref.	/
	**1–12 months**	1210	911	1.07	0.97–1.18	0.1484
	**13–24 months**	457	164	0.88	0.75–1.04	0.1399
	**25–36 months**	283	78	0.93	0.71–1.21	0.5697
	**>36 months**	503	71	1.15	0.86–1.54	0.3336
	**Trend (12 months)**			1.00	0.96–1.05	0.8403
Selective β-blockers						
	**0 DDDs**	1854	1113	1.00	Ref.	/
	**>0–365 DDDs**	1285	857	1.00	0.92–1.10	0.9515
	**366+ DDDs**	750	181	0.98	0.83–1.17	0.8455
	**Trend (180 DDDs)**			0.99	0.96–1.02	0.5039
	**0 months**	1929	1126	1.00	Ref.	/
	**1–12 months**	1104	809	1.04	0.94–1.15	0.4316
	**13–24 months**	421	150	**0.83**	**0.70–0.98**	**0.0312**
	**25–36 months**	253	68	0.95	0.72–1.25	0.6991
	**>36 months**	459	68	1.18	0.89–1.57	0.2570
	**Trend (12 months)**			1.00	0.96–1.04	0.9655
Nonselective β-blockers						
	**0 DDDs**	3622	1993	1.00	Ref.	/
	**>0–365 DDDs**	190	141	**1.33**	**1.11–1.59**	**0.0017**
	**366+ DDDs**	78	18	0.96	0.63–1.45	0.8445
	**Trend (180 DDDs)**			1.01	0.95–1.08	0.6488
	**0 months**	3864	2051	1.00	Ref.	/
	**1–12 months**	191	146	1.33	1.12–1.59	0.0015
	**13–24 months**	41	12	1.19	0.80–1.77	0.3975
	**25–36 months**	32	10	1.09	0.61–1.96	0.7720
	**>36 months**	39	2	0.63	0.21–1.93	0.4217
	**Trend (12 months)**			1.04	0.93–1.16	0.4599
Hydrophilic β-blockers						
	**0 DDDs**	3337	1867	1.00	Ref.	/
	**>0–365 DDDs**	346	241	1.08	0.95–1.24	0.2455
	**366+ DDDs**	199	41	0.83	0.61–1.13	0.2363
	**Trend (180 DDDs)**			0.99	0.94–1.03	0.6040
	**0 months**	3557	1930	1.00	Ref.	/
	**1–12 months**	333	230	1.04	0.90–1.21	0.5897
	**13–24 months**	92	34	0.98	0.74–1.30	0.8727
	**25–36 months**	77	17	0.93	0.59–1.48	0.7673
	**>36 months**	108	10	0.80	0.45–1.42	0.4488
	**Trend (12 months)**			0.98	0.91–1.05	0.4836
Lipophilic β-blockers						
	**0 DDDs**	2136	1264	1.00	Ref.	/
	**>0–365 DDDs**	1147	742	1.02	0.93–1.12	0.6444
	**366 + DDDs**	614	148	1.02	0.85–1.23	0.8111
	**Trend (180 DDDs)**			1.00	0.97–1.02	0.7671
	**0 months**	2227	1272	1.00	Ref.	/
	**1–12 months**	971	706	1.08	0.98–1.20	0.1062
	**13–24 months**	375	129	0.86	0.72–1.02	0.0906
	**25–36 months**	216	56	0.92	0.69–1.23	0.5889
	**>36 months**	377	58	1.22	0.92–1.63	0.1726
	**Trend (12 months)**			1.02	0.97–1.06	0.4855

Abbreviations: ATC = Anatomical Therapeutic Chemical Code, CI = Confidence interval, HR = Hazard ratio, PY = Person-years.

^a^Hazard ratio from Cox proportional hazard model on the propensity score matched groups with additional adjustment for time-dependent use of Non-steroidal anti-inflammatory drugs, statins, antihypertensive (other than β-blocker) and diabetes medication after diagnosis.

In general, also dose and duration specific analyses did not show much evidence for an association. An increase in mortality for doses between 0–365 DDDs of nonselective β-blockers was observed (1.33 (1.11–1.59)), whereas the HR for a cumulative duration of 13–24 months of selective β-blocker use (0.83 (0.70–0.98)) was decreased.

Comparing β-blocker use to non-users led again to much higher estimates as presented in Supplementary Table 4.

## Discussion

In this so far largest population-based study addressing the association of β-blocker use and lung cancer survival, we found no clinically relevant evidence for a survival benefit of pre- or post-diagnostic β-blocker use among lung cancer patients. There were some significant associations when stratifying for β-blocker subtypes, stage, site, dose or duration of use but they did not follow a consistent direction.

Results from previous studies on the association of β-blocker use and lung cancer survival were inconclusive and some of them are suspected to have reported too overoptimistic results which might be due to factors like immortal time bias^14^. So far, two studies have suggested a beneficial use of β-blockers for NSCLC patients. The latest study from 2015 suggested a 22% decrease in mortality, but was only based on hospital-based data from 673 patients with stage III NSCLC^9^. Another study by the same authors from 2013 showed similar results for distant metastasis-free survival, disease-free survival, and overall survival^10^.

In contrast, two other studies on lung cancer survival rather proposed no association between β-blockers and overall survival^7, 8^. However, also these two studies with a total of 107 and 435 patients, respectively, were both hospital-based and therefore limited to data ascertained from monocentric medical chart reviews. Additionally, in a recently published meta-analysis focusing on β-blocker use and cancer prognosis with special emphasis on immortal time bias, these studies were both suspected to have incorporated immortal person-time which further limits the interpretability of their findings^14^. Furthermore, two population-based screening studies including lung cancer patients reported no association between β-blocker use and overall survival after lung cancer with hazard ratios comparable to ours (1.01 (0.93–1.11) and 1.12 (0.89–1.41), respectively)^11, 12^.

All of our main results in both pre- and post-diagnostic analyses very precisely and consistently suggest that β-blocker use might not be associated to overall survival among lung cancer patients. However, subgroup analyses showed some inconsistent significant results which might be due to the following reasons.

Firstly, it is very likely that the majority of these results might be due to chance and distorted by other factors like small subgroup sample sizes and residual confounding caused by unmeasured covariates. Without adjustment for confounding, we generally observed higher HRs which decreased after comprehensive covariate adjustment in a sensitivity analysis (data not shown). Secondly, we performed a large number of statistical tests in stratified analyses which always creates the potential of statistical significance due to chance. The fact that we observed these significant results in both directions strengthens this assumption. Thirdly, many of these inconsistent findings were calculated in subgroups with low sample sizes like nonselective β-blockers, which are only rarely prescribed in clinical practice for a longer period of time.

### Strengths and limitations

This study has potential limitations. Although our calculations are based on a comprehensive database, we still excluded some patients with missing information, mainly on variables containing data on co-morbidity. We also refrained from imputing our missing data to conduct a complete case analysis. Coding patients with missing information on comorbidity as having no comorbidity in sensitivity analysis, however, did not alter the results (data not shown).

Additionally, antiangiogenic mechanisms were proposed to be mediated via β_2_-adrenoceptor blockade suppressing cAMP levels and activating extracellular signal-regulated kinase (EKR)1/2 in a dose dependent way^6^. However, our sample sizes for analyses involving only nonselective β-blockers, which are able to block β_2_-adrenoceptors, was small compared to the ones with β_1_ selective β-blockers.

Another limitation is the use of overall survival as our main outcome. As we had no information on the cause of death, we were not able to calculate lung cancer-specific survival or to investigate competing risks. However, given the poor prognosis of a lung cancer diagnosis, it is very likely that the majority of patients died due to their lung cancer disease. Nevertheless, overall survival needs to be interpreted carefully on the basis of the underlying medication use and co-morbidities. We addressed this problem by restricting the reference group to active comparators which led to equal cohorts in terms of health-seeking behaviors and frailty and therapy adherence. Additionally we accounted for this by adjusting for comorbidities, time-varying treatment and distinct numbers of medications used, as this was shown to have a good performance as a comorbidity measure to control for confounding^26^. Unfortunately, dose-responses could only be approximated by DDDs as no operationalisable variable was available to measure real drug utilization. This information could have also been of interest for assessment of therapy compliance.

However, our study also has unique strengths. To our knowledge this is the largest and most comprehensive population-based cohort study which has been conducted so far focusing on the association of β-blocker use and lung cancer mortality. A power analysis indicated that a harmful or protective association with HRs ≥ 1.13 or HRs ≤ 0.89, respectively, could have been detected in our main analysis with a significance level of α = 5% and a power of 80% (β = 0.2)^33^. For subgroup analyses only stronger associations could have been detected, however, (e.g. HR ≥ 1.45/≤0.69, ≥1.70/≤0.59, ≥1.24/≤0.81, ≥1.20/≤0.83 in stage specific analyses for stage I, II, III and IV cancers, respectively). Nevertheless, we were able to address key exposure characteristics, such as tissue availability of β-blockers (influenced by physico-chemical properties such as lipophilicity), pharmacodynamic characteristics (receptor selectivity), cumulative treatment duration, and dose as well as key tumor characteristics such as stage and histological subtypes. Also tumor stage was addressed as an important factor because angiogenic markers appear to have more prognostic value in earlier stages of the disease^34^ and probably rather in NSCLC than in SCLC^35^.

In contrast to the majority of the previously published studies, our analysis was based on data from a population-based cohort, which allows for a more generalizable interpretation of the association of β-blocker use and lung cancer prognosis than in monocentric hospital-acquired data. In addition to the high quality and validity of the underlying data we were able to conduct a comprehensive confounder adjustment, which was shown to be of outstanding importance in pharmacoepidemiological studies^15, 36^.

Because several previous studies did not consider time-dependent treatment effects, making them prone to a variety of biases, especially immortal time bias^14^, we carefully addressed this issue by modelling post-diagnostic β-blocker use according to the Mantel-Byar method as simulations studies demonstrated that this is the gold-standard for time-dependent modelling^13, 21^.

As shown in previous studies, it is also of importance to address paradoxical relations of drug treatment in elderly populations in terms of sick-stopper effects, which means that moribund patients are likely to discontinue preventive medication for, in this case, non-lung cancer related conditions^22^. Hence, a strength of our analysis is that we accounted for these end-of-life treatment effects with a first-treatment-carried-forward approach (intention-to-treat analysis) and an additional four-month lag after a patient’s first β-blocker dispensing to avoid spurious findings due to reverse causality and informative censoring.

### Conclusion and implications for clinical practice

Βeta-blockers are well-established and indisputably valuable drugs for managing cardiovascular diseases, hypertension, heart failure, primary migraine prophylaxis, and essential tremor. The effectiveness of propranolol in the treatment of infantile hemangiomas, which is assumed to be based on antiangiogenic actions mediated by the β_2_-adrenoceptor^6^, might encourage further studies to concentrate on larger patient populations receiving non-selective β-blocker therapy, tumor entities and stages^34^ for which angiogenesis has a high impact on tumor progression^37–39^. Utilization of β-blockers as an add-on therapy in cancer treatment would be of utmost interest not only to inhibit metastatic spread and cell growth, but also for public health implications. However, our study does not support such use for patients with lung cancer.

In conclusion, after careful interpretation of our data and taking further sensitivity analyses and possible residual confounding into account, we did not find evidence for a beneficial role of pre- or post-diagnostic β-blocker use for lung cancer patients in this so far largest and most comprehensive study. More evidence and a final conclusion will have to come from currently ongoing randomized clinical trials^40–44^.

## Electronic supplementary material

Supplementary Material
