# Supplementary material for: Pre- and post-diagnostic β-blocker use and lung cancer survival: A population-based cohort study
Source: Sci Rep. 2017 Jun 6;7:2911. doi: 10.1038/s41598-017-02913-8 (PMC5460218; doi:10.1038/s41598-017-02913-8)
Supplement: Supplementary file 1 — Supplementary Material [file 41598_2017_2913_MOESM1_ESM.pdf]

SUPPLEMENTARY MATERIAL

Pre- and post-diagnostic β-blocker use and lung cancer survival: A population-based cohort study

Janick Weberpals[1], RPh, Lina Jansen[1], PhD, Walter E. Haefeli[2], MD, Michael Hoffmeister[1], PhD, Martin Wolkewitz[3], PhD, Myrthe P. P. van Herk-Sukel[4], PhD, Pauline A.J. Vissers[5], PhD, Hermann Brenner*[1,6,7], MD, MPH

[1] Division of Clinical Epidemiology and Aging Research, German Cancer Research Center (DKFZ), Heidelberg, Germany

[2] Department of Clinical Pharmacology and Pharmacoepidemiology, University Hospital of Heidelberg, Heidelberg, Germany

[3] Center for Medical Biometry and Medical Informatics, Institute for Medical Biometry and Statistics, Medical Center, University of Freiburg, Germany

[4] PHARMO Institute for Drug Outcomes Research, Utrecht, The Netherlands

[5] Netherlands Comprehensive Cancer Organisation, Utrecht, The Netherlands

[6] Division of Preventive Oncology, National Center for Tumor Diseases (NCT), German Cancer Research Center (DKFZ), Heidelberg, Germany

[7] German Cancer Consortium (DKTK), German Cancer Research Center (DKFZ), Heidelberg, Germany

***Correspondence to** Hermann Brenner, Division of Clinical Epidemiology and Aging Research, German Cancer Research Center (dkfz), Im Neuenheimer Feld 581, 69120 Heidelberg, Germany, Phone: +49-6221-42-1300; Fax: +49-6221-42-1302; E-mail: h.brenner@dkfz.de

SUPPLEMENTARY TABLES

Supplementary Table 1. Classification of Medication Classes according to ATC codes of the Dispensings

Drug class	International Nonproprietary Name (INN)/Subclass	ATC Codes
**β-blockers**		
Any β-blocker	-	Starting with 'C07'
Selective β-blocker (according WHO/ATC coding)	Metoprolol, Atenolol, Acebutolol, Bisoprolol, Celiprolol, Esmolol, Nebivolol	C07AB<br>Combinations: C07BB, C07CB, C07DB, C07EB, C07FB
Nonselective β-blocker (according WHO/ATC coding)	Alprenolol, Oxprenolol, Pindolol, Propranolol, Timolol, Sotalol	C07AA<br>Combinations: C07BA, C07CA, C07DA, C07EA, C07FA
Alpha and β-blocking agents	Carvedilol, Laβlol	C07AG<br>Combinations: C07BG, C07CG
Lipophilic character	Propranolol, Nebivolol, Carvedilol, Alprenolol, Bisoprolol, Metoprolol, Pindolol, Acebutolol, Timolol (systemic)	See single active ingredients
Hydrophilic character	Atenolol, Sotalol, Nadolol	See single active ingredients
<u>**Single active ingredients (and combinations)**</u>		
Metoprolol	C07AB02, C07FB02, C07CB02, C07BB02, C07BB52, C07AB52	
Bisoprolol	C07AB07, C07FB07, C07BB07, C07AB57, C09BX02	
Carvedilol	C07AG02	
Atenolol	C07AB03, C07FB03, C07CB03, C07CB53, C07BB03, C07DB01, C07AB11	
Propranolol	C07AA05, C07FA05, C07BA05	
Nebivolol	C07AB12, C07FB12, C07BB12	
Sotalol	C07AA07, C07BA07, C07AA57	
Timolol (systemic)	C07AA06, C07AA06, C07BA06, C07DA06	
Pindolol	C07AA17, C07CA17, C07AA14, C07AA03, C07CA03	
Acebutolol	C07AB04, C07BB04	
**Other antihypertensive treatment**	C02, C03, C04, C08, C09	
**Statins**	C10AA, C10BA, C10BX	
**Non-steroidal anti-inflammatory drugs (NSAIDs)**	M01A, M01B, N02BA, B01AC06, B01AC30, B01AC34, B01AC36, B01AC56, B01AC30	
**Diabetes medication**	A10	
**Comparison group (medications used for same indications as β-blockers)[a]**	Coronary Heart Disease & Acute Myocardial Infarction	C01DA, C08, C01EB17, C01DX16, C01EB15, C01EB18,<br>C01DX12, C10A, C10B, B01AC
	Congestive Heart Failure	C09A, C09B, C09C, C09D, C03A, C03B, C03C, C03D<br>C03E, C02D, C01A
	Cardiac Arrhythmias	C01B, C08D, B01AA, B01AE<br>B01AF, C01BG11, C01EB10
	Hypertension	C09X, C02A, C02CA, C02LA<br>C02LB, C02LC, C02LE, C02LF<br>C02LG, C02LK

[a] If medications are prescribed for more than one indication, they are listed only once.

Supplementary Table 2. Association between Pre-diagnostic β-blocker use and Lung Cancer Survival (Overall, by Cancer Site and Cancer Stage) when Comparing to Non-use.*

β-blocker/ subgroup	All eligible patients (N=6172)					Propensity score matched cohort (N=2874)				
	N	Events	HR[a]	95% CI	P	N	Events	HR[b]	95% CI	P
**No β-blocker**										
**Total**	4735	4120	1.00	Ref.	/	1437	1280	1.00	Ref.	/
**Stage I**	713	414	1.00	Ref.	/	229	143	1.00	Ref.	/
**Stage II**	266	171	1.00	Ref.	/	80	63	1.00	Ref.	/
**Stage III**	1348	1229	1.00	Ref.	/	412	384	1.00	Ref.	/
**Stage IV**	1907	1847	1.00	Ref.	/	538	525	1.00	Ref.	/
**NSCLC**	3458	2919	1.00	Ref.	/	1014	879	1.00	Ref.	/
**SCLC**	814	773	1.00	Ref.	/	249	240	1.00	Ref.	/
**Any β-blocker**										
**Total**	1437	1265	0.99	0.92-1.07	0.8114	1437	1265	1.00	0.93-1.09	0.9325
**Stage I**	239	152	1.06	0.84-1.33	0.6261	239	152	1.03	0.82-1.29	0.8139
**Stage II**	79	65	1.03	0.70-1.52	0.8629	79	65	0.99	0.69-1.42	0.9631
**Stage III**	411	366	**0.86**	**0.75-0.98**	**0.0278**	411	366	0.92	0.79-1.06	0.2540
**Stage IV**	549	539	1.08	0.96-1.21	0.2125	549	539	1.11	0.99-1.25	0.0797
**NSCLC**	1027	883	0.98	0.90-1.07	0.7142	1027	883	0.99	0.90-1.09	0.7871
**SCLC**	252	239	1.13	0.95-1.34	0.1541	252	239	1.14	0.95-1.36	0.1493
**Selective β-blocker**										
**Total**	1278	1122	0.98	0.91-1.05	0.5515	1278	1122	0.97	0.90-1.06	0.5212
**Stage I**	215	139	1.12	0.89-1.41	0.3208	215	139	1.08	0.86-1.37	0.5098
**Stage II**	69	56	0.97	0.65-1.47	0.8989	69	56	0.96	0.66-1.41	0.8543
**Stage III**	368	327	**0.85**	**0.74-0.98**	**0.0277**	368	327	0.90	0.77-1.04	0.1556
**Stage IV**	484	474	1.07	0.95-1.21	0.2395	484	474	1.07	0.95-1.21	0.2447
**NSCLC**	909	781	0.99	0.91-1.09	0.8636	909	781	0.98	0.88-1.08	0.6280
**SCLC**	229	216	1.09	0.91-1.30	0.3482	229	216	1.04	0.87-1.25	0.6517
**Nonselective β-blocker**										
**Total**	178	162	1.08	0.92-1.27	0.3488	178	162	**1.19**	**1.01-1.41**	**0.0398**
**Stage I**	26	15	0.81	0.47-1.40	0.4516	26	15	0.84	0.48-1.47	0.5350
**Stage II**	10	9	1.25	0.60-2.58	0.5520	10	9	1.11	0.59-2.06	0.7536
**Stage III**	53	49	0.99	0.73-1.33	0.9409	53	49	1.22	0.90-1.65	0.2015
**Stage IV**	70	70	1.00	0.79-1.28	0.9759	70	70	1.14	0.91-1.43	0.2498
**NSCLC**	131	115	0.98	0.81-1.19	0.8428	131	115	1.09	0.90-1.33	0.3745
**SCLC**	25	25	1.22	0.81-1.84	0.3461	25	25	**1.67**	**1.16-2.40**	**0.0062**
**Hydrophilic β-blocker**										
**Total**	349	307	**0.87**	**0.77-0.98**	**0.0256**	349	307	0.98	0.87-1.10	0.7142
**Stage I**	55	40	1.23	0.87-1.73	0.2442	55	40	1.36	0.95-1.95	0.0884
**Stage II**	25	23	1.04	0.63-1.70	0.8849	25	23	1.26	0.80-1.99	0.3124
**Stage III**	89	73	**0.72**	**0.56-0.92**	**0.0084**	89	73	0.82	0.63-1.08	0.1643
**Stage IV**	134	132	0.90	0.75-1.09	0.2841	134	132	1.01	0.85-1.22	0.8814
**NSCLC**	234	203	0.92	0.80-1.07	0.2961	234	203	1.01	0.87-1.17	0.9306
**SCLC**	67	63	0.94	0.71-1.23	0.6312	67	63	1.06	0.80-1.40	0.6802
**Lipophilic β-blocker**										
**Total**	1073	943	1.06	0.98-1.14	0.1669	1073	943	1.01	0.93-1.10	0.8280
**Stage I**	184	111	0.94	0.74-1.21	0.6467	184	111	0.88	0.70-1.110	0.2814
**Stage II**	53	41	0.98	0.65-1.47	0.9163	53	41	0.83	0.56-1.21	0.3335
**Stage III**	320	291	0.95	0.82-1.10	0.4936	320	291	1.00	0.86-1.16	0.9998
**Stage IV**	405	397	**1.15**	**1.01-1.30**	**0.0294**	405	397	1.11	0.98-1.26	0.0929

β-blocker/ subgroup	All eligible patients (N=6172)					Propensity score matched cohort (N=2874)				
	N	Events	HR[a]	95% CI	P	N	Events	HR[b]	95% CI	P
**NSCLC**	782	669	1.03	0.93-1.13	0.6013	782	669	0.98	0.89-1.09	0.7449
**SCLC**	181	172	1.14	0.95-1.38	0.1696	181	172	1.08	0.89-1.30	0.4499

Abbreviations: ATC=Anatomical Therapeutic Chemical Code, CI=Confidence interval, HR=Hazard ratio, NSAIDs=Non-steroidal anti-inflammatory drugs, NSCLC=Non-small cell lung cancer, PY=Person-years, SCLC=Small cell lung cancer

[a] Hazard ratio from the Cox proportional hazard model β-blocker use four months prior diagnosis; adjusted for age, sex, year of diagnosis, socio-economic status, comorbidities (cardiovascular, hypertension, cerebrovascular, lung, diabetes) , treatment (surgery, chemotherapy, radiotherapy, radiotherapy aimed at metastasis), best supportive care, stage, histology (NSCLC, SCLC, Other), previous cancer, time-dependent use of NSAIDs, statins, antihypertensive (other than β-blocker) and diabetes medication after diagnosis and number of distinct ATC classes prescribed during four months prior to diagnosis. Stratification factors were omitted from the stratified models.

[b] Hazard ratio from the Cox proportional hazard model for β-blocker use four months prior diagnosis; adjusted for time-dependent use of NSAIDs, statins, antihypertensive (other than β-blocker) and diabetes medication.

Supplementary Table 3. Association between <u>Post-diagnostic β-blocker use</u> and Lung Cancer Survival (Overall, by Histological Subtype and Cancer Stage) when Comparing to Non-use.*

β-blocker/ subgroup	All eligible patients (N=6172)					Propensity score matched cohort (N=2874)				
	PY	Events	HR[a]	95% CI	P	PY	Events	HR[b]	95% CI	P
**<u>No β-blocker</u>**										
**Total**	7738	3800	1.00	Ref.	/	1944	1132	1.00	Ref.	/
**Stage I**	2575	349	1.00	Ref.	/	652	113	1.00	Ref.	/
**Stage II**	773	155	1.00	Ref.	/	170	58	1.00	Ref.	/
**Stage III**	1975	1107	1.00	Ref.	/	489	329	1.00	Ref.	/
**Stage IV**	1677	1766	1.00	Ref.	/	392	488	1.00	Ref.	/
**NSCLC**	6525	2678	1.00	Ref.	/	1483	880	1.00	Ref.	/
**SCLC**	950	723	1.00	Ref.	/	237	240	1.00	Ref.	/
**<u>Any β-blocker</u>**										
**Total**	3453	1585	**1.09**	**1.02-1.17**	**0.0148**	2855	1413	1.03	0.95-1.12	0.4430
**Stage I**	1375	217	1.08	0.88-1.33	0.4492	1111	182	1.01	0.79-1.28	0.9429
**Stage II**	336	81	1.06	0.75-1.49	0.7560	226	70	0.99	0.69-1.42	0.9510
**Stage III**	908	488	1.00	0.88-1.14	0.9990	777	421	0.96	0.83-1.12	0.6061
**Stage IV**	474	620	**1.13**	**1.02-1.26**	**0.0257**	440	576	1.11	0.99-1.25	0.0851
**NSCLC**	2824	1124	1.08	0.99-1.17	0.0835	2295	985	0.99	0.90-1.09	0.8799
**SCLC**	312	289	**1.26**	**1.07-1.48**	**0.0057**	286	268	**1.22**	**1.02-1.46**	**0.0279**
**<u>Selective β-blocker</u>**										
**Total**	3079	1404	**1.08**	**1.00-1.16**	**0.0379**	2569	1260	1.01	0.93-1.09	0.8848
**Stage I**	1204	192	1.15	0.94-1.42	0.1834	978	164	1.07	0.84-1.35	0.5886
**Stage II**	297	73	1.13	0.79-1.62	0.5152	200	63	1.03	0.71-1.50	0.8807
**Stage III**	817	439	1.01	0.88-1.14	0.9365	707	382	0.96	0.82-1.12	0.5941
**Stage IV**	422	542	**1.13**	**1.01-1.26**	**0.0370**	395	508	1.07	0.95-1.20	0.2967
**NSCLC**	2490	995	**1.10**	**1.01-1.19**	**0.0321**	2040	882	1.00	0.91-1.11	0.9492
**SCLC**	293	258	**1.19**	**1.01-1.41**	**0.0425**	268	239	1.09	0.91-1.30	0.3626
**<u>Nonselective β-blocker</u>**										
**Total**	519	241	**1.17**	**1.02-1.33**	**0.0226**	400	205	**1.21**	**1.04-1.41**	**0.0143**
**Stage I**	218	34	0.97	0.68-1.39	0.8748	164	25	0.96	0.64-1.45	0.8496
**Stage II**	53	15	1.36	0.76-2.44	0.2956	32	12	1.26	0.71-2.25	0.4346
**Stage III**	150	80	1.08	0.86-1.37	0.5123	123	67	1.18	0.90-1.55	0.2311
**Stage IV**	60	87	1.04	0.84-1.30	0.7074	51	76	1.18	0.95-1.45	0.1296
**NSCLC**	455	175	1.06	0.90-1.23	0.4941	354	143	1.08	0.90-1.28	0.4204
**SCLC**	28	39	1.33	0.95-1.85	0.0930	24	35	**1.76**	**1.29-2.38**	**0.0003**
**<u>Hydrophilic β-blocker</u>**										
**Total**	895	383	0.93	0.84-1.04	0.2035	743	342	1.00	0.89-1.12	0.9450
**Stage I**	288	56	1.19	0.89-1.60	0.2314	232	48	1.34	0.98-1.83	0.0670
**Stage II**	103	28	1.01	0.64-1.59	0.9661	60	24	1.32	0.84-2.09	0.2295
**Stage III**	237	103	0.83	0.68-1.03	0.0902	210	89	0.88	0.68-1.14	0.3226
**Stage IV**	116	149	0.94	0.79-1.12	0.4660	106	136	1.02	0.86-1.23	0.7873
**NSCLC**	674	261	0.97	0.85-1.11	0.6525	542	227	1.01	0.87-1.16	0.9239
**SCLC**	89	74	1.02	0.79-1.31	0.9008	84	69	1.11	0.85-1.45	0.4569
**<u>Lipophilic β-blocker</u>**										
**Total**	2733	1228	1.15	1.07-1.24	0.0002	2246	1092	1.03	0.95-1.12	0.4779
**Stage I**	1154	169	0.99	0.80-1.22	0.9111	924	142	0.89	0.71-1.13	0.3376
**Stage II**	250	59	1.21	0.84-1.73	0.3084	169	49	0.88	0.61-1.28	0.5169
**Stage III**	729	400	1.09	0.96-1.24	0.1979	619	345	1.04	0.89-1.21	0.6046
**Stage IV**	358	465	**1.19**	**1.06-1.33**	**0.0041**	336	435	1.10	0.97-1.24	0.1435

β-blocker/ subgroup	All eligible patients (N=6172)					Propensity score matched cohort (N=2874)				
	PY	Events	HR[a]	95% CI	P	PY	Events	HR[b]	95% CI	P
**NSCLC**	2287	883	**1.12**	**1.03-1.22**	**0.0112**	1849	773	0.99	0.90-1.09	0.8658
**SCLC**	234	217	**1.23**	**1.04-1.47**	**0.0171**	214	201	1.14	0.95-1.38	0.1530

Abbreviations: ATC=Anatomical Therapeutic Chemical Code, CI=Confidence interval, HR=Hazard ratio, NSAIDs=Non-steroidal anti-inflammatory drugs, NSCLC=Non-small cell lung cancer, PY=Person-years, SCLC=Small cell lung cancer

[a] Hazard ratio from the Cox proportional hazard model for time-dependent β-blocker use; adjusted for age, sex, year of diagnosis, socio-economic status, comorbidities (cardiovascular, hypertension, cerebrovascular, lung, diabetes) , treatment (surgery, chemotherapy, radiotherapy, radiotherapy aimed at metastasis), best supportive care, stage, histology (NSCLC, SCLC, Other), previous cancer, time-dependent use of NSAIDs, statins, antihypertensive (other than β-blocker) and diabetes medication after diagnosis and number of distinct ATC classes prescribed during four months prior to diagnosis. Stratification factors were omitted from the stratified models.

[b] Hazard ratio from the Cox proportional hazard model for time-dependent β-blocker use; adjusted for time-dependent use of NSAIDs, statins, antihypertensive (other than β-blocker) and diabetes medication.

Supplementary Table 4. Association between Post-diagnostic Cumulative Dose and Cumulative Duration of β-blocker use and Overall Lung Cancer Survival (Overall, by β-receptor Affinity and Pharmacokinetic Characteristics) when Comparing to Non-use.*

β-blocker/Exposure	All eligible patients (N=6172)					Propensity score matched cohort (N=2874)				
	PY	Events	HR[a]	95% CI	P	PY	Events	HR[b]	95% CI	P
**Any β-blocker**										
**0 DDDs**	7555	3778	1.00	Ref.	/	1864	1123	1.00	Ref.	/
**>0 - 365 DDDs**	2047	1223	**1.08**	**1.00-1.16**	**0.0542**	1625	1093	1.04	0.95-1.13	0.4214
**366+ DDDs**	1077	251	0.98	0.85-1.13	0.7492	962	232	0.98	0.84-1.15	0.8337
**Trend (180 DDDs)**			1.00	0.97-1.02	0.7726			0.99	0.97-1.02	0.4667
**0 months**	7738	3800	1.00	Ref.	/	1944	1132	1.00	Ref.	/
**1-12 months**	1799	1178	**1.14**	**1.05-1.23**	**0.0014**	1410	1047	1.08	0.98-1.18	0.1197
**13-24 months**	627	213	0.94	0.83-1.07	0.3658	543	193	0.92	0.79-1.07	0.2698
**25-36 months**	375	103	0.97	0.79-1.20	0.8120	331	96	0.94	0.74-1.20	0.6430
**> 36 months**	652	91	**1.28**	**1.04-1.57**	**0.0224**	571	77	1.06	0.80-1.39	0.6932
**Trend (12 months)**			**1.04**	**1.01-1.08**	**0.0124**			1.01	0.97-1.05	0.7977
**Selective β-blockers**										
**0 DDDs**	7908	3948	1.00	Ref.	/	2129	1268	1.00	Ref.	/
**>0 - 365 DDDs**	1822	1089	**1.07**	**1.00-1.16**	**0.0665**	1475	981	1.01	0.93-1.1	0.8052
**366+ DDDs**	966	218	0.95	0.81-1.10	0.4750	853	201	0.95	0.8-1.11	0.4966
**Trend (180 DDDs)**			0.99	0.97-1.02	0.5299			0.99	0.96-1.01	0.3173
**0 months**	8112	3981	1.00	Ref.	/	2230	1285	1.00	Ref.	/
**1-12 months**	1585	1037	**1.14**	**1.05-1.23**	**0.0017**	1267	927	1.05	0.96-1.16	0.2808
**13-24 months**	567	191	0.89	0.77-1.02	0.0820	496	176	0.86	0.73-1.00	0.0514
**25-36 months**	340	90	0.98	0.79-1.21	0.8301	297	85	0.98	0.76-1.25	0.8462
**> 36 months**	587	86	1.27	1.02-1.57	0.0306	509	72	1.10	0.84-1.44	0.5024
**Trend (12 months)**			**1.04**	**1.00-1.08**	**0.0286**			1.00	0.96-1.04	0.9009
**Nonselective β-blockers**										
**0 DDDs**	1022	5032	1.00	Ref.	/	4104	2260	1.00	Ref.	/
**>0 - 365 DDDs**	350	198	**1.20**	**1.04-1.39**	**0.0131**	244	168	**1.29**	**1.10-1.52**	**0.0018**
**366+ DDDs**	115	25	0.83	0.56-1.23	0.3542	112	23	0.86	0.61-1.22	0.4004
**Trend (180 DDDs)**			1.02	0.97-1.07	0.5301			1.01	0.96-1.06	0.6810
**0 months**	1067	5144	1.00	Ref.	/	4399	2340	1.00	Ref.	/
**1-12 months**	345	204	**1.19**	**1.02-1.38**	**0.0260**	250	175	**1.28**	**1.09-1.51**	**0.0033**
**13-24 months**	78	20	1.12	0.83-1.52	0.4680	56	15	1.16	0.83-1.63	0.3842
**25-36 months**	40	13	1.05	0.58-1.90	0.8803	37	11	1.00	0.57-1.77	0.9886
**> 36 months**	57	4	1.12	0.56-2.27	0.7476	57	4	0.64	0.32-1.28	0.2109
**Trend (12 months)**			1.07	0.99-1.15	0.1056			1.03	0.95-1.13	0.4535
**Hydrophilic β-blockers**										
**0 DDDs**	9861	4882	1.00	Ref.	/	3774	2118	1.00	Ref.	/
**>0 - 365 DDDs**	569	318	1.01	0.90-1.13	0.8772	431	281	1.06	0.93-1.20	0.3682
**366+ DDDs**	250	52	0.63	0.47-0.83	0.0010	245	49	0.79	0.60-1.04	0.0946
**Trend (180 DDDs)**			0.95	0.92-0.99	0.0262			0.99	0.95-1.03	0.6129
**0 months**	1029	5002	1.00	Ref.	/	4056	2203	1.00	Ref.	/
**1-12 months**	520	303	1.00	0.88-1.14	0.9609	407	271	1.03	0.90-1.18	0.6307
**13-24 months**	149	43	0.82	0.65-1.04	0.0996	122	38	0.98	0.76-1.26	0.8487
**25-36 months**	98	23	0.76	0.50-1.17	0.2129	88	20	0.86	0.56-1.33	0.5013
**> 36 months**	128	14	0.78	0.51-1.18	0.2368	127	13	0.80	0.49-1.30	0.3742

β-blocker/Exposure	All eligible patients (N=6172)					Propensity score matched cohort (N=2874)				
	**PY**	**Events**	**HR[a]**	**95% CI**	**P**	**PY**	**Events**	**HR[b]**	**95% CI**	**P**
**Trend (12 months)**			0.95	0.89-1.01	0.0925			0.98	0.92-1.04	0.5307
**Lipophilic β-blockers**										
**0 DDDs**	8247	4133	1.00	Ref.	/	2444	1442	1.00	Ref.	/
**>0 - 365 DDDs**	1650	946	1.12	1.04-1.21	0.0046	1322	848	1.02	0.94-1.12	0.6032
**366+ DDDs**	809	179	1.08	0.92-1.27	0.3432	701	163	0.98	0.82-1.16	0.7953
**Trend (180 DDDs)**			1.02	0.99-1.04	0.2409			0.99	0.96-1.02	0.4521
**0 months**	8458	4157	1.00	Ref.	/	2553	1453	1.00	Ref.	/
**1-12 months**	1428	912	**1.20**	**1.11-1.30**	**<0.0001**	1121	807	1.08	0.98-1.19	0.1045
**13-24 months**	512	167	0.95	0.83-1.10	0.5219	446	154	0.88	0.75-1.03	0.1001
**25-36 months**	298	76	1.03	0.82-1.29	0.8077	256	70	0.95	0.74-1.23	0.7136
**> 36 months**	495	73	**1.50**	**1.20-1.89**	**0.0004**	423	61	1.13	0.86-1.48	0.3833
**Trend (12 months)**			**1.08**	**1.04-1.12**	**0.0001**			1.01	0.97-1.06	0.5823

Abbreviations: ATC=Anatomical Therapeutic Chemical Code, CI=Confidence interval, HR=Hazard ratio, NSAIDs=Non-steroidal anti-inflammatory drugs, PY=Person-years

[a] Hazard ratio from the Cox proportional hazard model; adjusted for age, sex, year of diagnosis, socio-economic status, comorbidities (cardiovascular, hypertension, cerebrovascular, lung, diabetes) , treatment (surgery, chemotherapy, radiotherapy, radiotherapy aimed at metastasis), best supportive care, stage, histology (NSCLC, SCLC, Other), previous cancer, time-dependent use of NSAIDs, statins, antihypertensive (other than β-blocker) and diabetes medication after diagnosis and number of distinct ATC classes prescribed during four months prior to diagnosis. Stratification factors were omitted from the stratified models.

[b] Hazard ratio from the Cox proportional hazard model for time-dependent β-blocker use; adjusted for time-dependent use of NSAIDs, statins, antihypertensive (other than β-blocker) and diabetes medication.
